# Robot-assisted nephrectomy for retroperitoneal liposarcoma compressing the kidney: a case report with urological management perspective

**DOI:** 10.1186/s12894-026-02186-2

**Published:** 2026-05-18

**Authors:** Hao Shen, Yuyong Wang, Zhanwen Dong, Jieting Wu

**Affiliations:** 1 The Fourth School of Clinical Medicine, Zhejiang Chinese Medical University, Hangzhou First People’s Hospital, Hangzhou, China; 2 Department of Urology, School Of Medicine, Affiliated Hangzhou First People’s Hospital, Westlake University, Hangzhou, China; 3 Baizhang Town Community Health Service Center, Hangzhou, China

**Keywords:** Retroperitoneal liposarcoma, Giant retroperitoneal liposarcoma, Robot-assisted surgery, Retroperitoneal approach, Nephrectomy, Case report

## Abstract

**Background:**

Liposarcoma (LS) is the second most common histological subtype of soft tissue sarcoma, accounting for approximately 7% of all soft tissue sarcomas, among which retroperitoneal liposarcoma (RPLS) is not a rare clinical entity. While RPLS involving the kidney and requiring combined urological management is relatively uncommon with limited clinical reports, it is often asymptomatic in the early stage, with clinical symptoms appearing only when the tumor compresses adjacent organs. Surgical resection is the cornerstone of treatment.

**Case presentation:**

A 54-year-old female was diagnosed with a well-differentiated giant retroperitoneal RPLS (250 mm × 92 mm) compressing the right kidney via contrast-enhanced MRI. After strict multidisciplinary evaluation in accordance with the 2024 NCCN Guidelines for Soft Tissue Sarcoma, preoperative core needle biopsy was not performed, and the retroperitoneal approach was selected for surgical intervention. On August 6, 2024, Da Vinci Xi Surgical System robot-assisted retroperitoneal tumor resection combined with right nephrectomy and retroperitoneal lymph node dissection was successfully completed via the retroperitoneal approach. Postoperative pathology and immunohistochemistry (MDM2 (+), S-100 (+), etc.) confirmed the diagnosis. The patient recovered uneventfully and was discharged on postoperative day 6 with individualized adjuvant targeted therapy. Follow-up CT scans at 3 months, 6 months, and 1 year postoperatively showed no tumor recurrence, and laboratory examinations such as serum creatinine were normal. This case preliminarily confirms the feasibility and safety of robot-assisted retroperitoneal approach for strictly selected giant RPLS (> 20 cm) confined to the retroperitoneal space, and provides a standardized urological management reference for similar cases.

**Discussion:**

Most existing robot-assisted retroperitoneal liposarcoma (RPLS) surgeries are performed via the transabdominal approach, and the reported cases of retroperitoneal approach are limited to moderately sized tumors (diameter < 10 cm). This case demonstrates the feasibility and safety of robot-assisted retroperitoneal approach for giant RPLS (> 20 cm) strictly confined to the retroperitoneal space, expands the application scope of this approach, and provides a standardized urological management strategy for RPLS compressing the kidney for clinical reference.

**Supplementary Information:**

The online version contains supplementary material available at 10.1186/s12894-026-02186-2.

## Background

Liposarcoma (LS) is the second most common histological subtype of soft tissue sarcoma, accounting for approximately 7% of all soft tissue sarcomas [1]. It most commonly occurs in areas rich in adipose tissue, such as the extremities and retroperitoneal space. RPLS itself is the most common primary retroperitoneal malignant tumor, accounting for more than 40% of all primary retroperitoneal malignant tumors, which is not a rare clinical entity; whereas RPLS involving the kidney or perirenal region accounts for less than 10% of all RPLS cases, with limited clinical reports focusing on its urological management strategies [2]. The retroperitoneal space contains loose connective tissue, which provides ample room for tumor expansion. Clinical symptoms typically appear only when the tumor becomes large enough to compress adjacent organs, and surgical resection with R0 margin remains the cornerstone of treatment.

Currently, robot-assisted surgery for RPLS is predominantly performed via the transabdominal approach, and the largest published case series of robot-assisted retroperitoneal approach for retroperitoneal tumors (105 patients) confirmed that the critical threshold for successful surgery was a maximum tumor diameter ≤ 64 mm [3]. All existing clinical studies on robot-assisted retroperitoneal approach for RPLS are limited to moderately sized tumors (diameter < 10 cm), with no published reports on its application in giant RPLS (> 20 cm) worldwide. Meanwhile, there is no standardized urological decision-making pathway for RPLS compressing the kidney, including preoperative biopsy, nephron-sparing surgery (NSS) assessment, and surgical approach selection.

In 2024, our department successfully performed Da Vinci Xi Surgical System-assisted resection of a 25 cm giant well-differentiated RPLS (WDL) compressing the right kidney, combined with right nephrectomy and retroperitoneal lymph node dissection via the retroperitoneal approach. The aim of this study was to verify the feasibility and safety of the robot-assisted retroperitoneal approach for strictly selected giant RPLS, and to establish a standardized urological management strategy for RPLS compressing the kidney. Follow-up CT scans at 3 months, 6 months, and 1 year after the operation revealed no evidence of local recurrence or metastatic lesions, indicating a favorable short-term outcome.

## Case presentation

### Patient information and preoperative evaluation

**Fig. 1:**
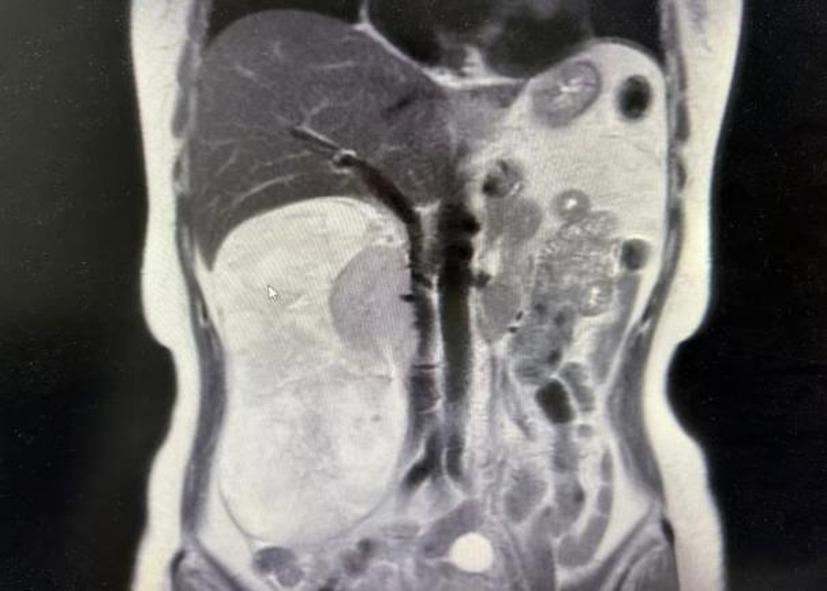
Contrast-enhanced MRI showing a giant retroperitoneal tumor measuring approximately 25.0 cm × 9.71 cm, compressing the adjacent normal right renal parenchyma

The patient was a 54-year-old female who presented with a right renal tumor identified by a routine physical examination 9 days prior. She reported no significant back pain, abdominal discomfort, distension, or other symptoms. Her appetite and general physical condition were normal, and she denied recent weight loss. Her medical history was unremarkable, with no significant comorbidities; family and psycho-social history, as well as relevant genetic information, were unremarkable. Physical examination revealed a flat, soft abdomen with no tenderness or palpable mass. Percussion tenderness over the right renal area was absent, and there was no tenderness along the right ureteral trajectory. Ultrasonography performed at a local hospital indicated a hyperechoic lesion in the right renal region, which was initially misdiagnosed as an intrarenal lipoma. Following admission, contrast-enhanced MRI of the kidneys revealed a heterogeneous mass measuring 250 mm × 92 mm in the right retroperitoneal space, containing fat components and demonstrating mild enhancement of solid portions, consistent with a well-differentiated LS. A small branch of the right renal artery was identified along the lesion margin. The right kidney was compressed and displaced, showing malrotation with lateral deviation of the renal hilum. During the excretory phase, two right renal pelves (consistent with a congenital duplex renal collecting system) were observed converging into the upper segment of the right ureter. A strip-like soft tissue density with tortuous veins was visible in the right adrenal region. The left kidney, adrenal gland and both ureters appeared normal (Fig. 1). The left renal pelvis and renal vessels were also unremarkable. Routine laboratory examination and comprehensive imaging findings led to the diagnosis of retroperitoneal well-differentiated liposarcoma (LS). Preoperative serum creatinine (Scr) was measured at 100µmol/L, and no urinary protein, red blood cells, or other abnormalities were detected on urinalysis.

### Preoperative multidisciplinary evaluation and decision-making

#### Preoperative biopsy decision-making

Preoperative core needle biopsy was not performed in this case, and the complete decision-making process and rationale are as follows: (1) The typical imaging features on contrast-enhanced MRI (dominant fat component, thick fibrous septa, and mild solid enhancement) were highly consistent with low-grade well-differentiated liposarcoma (WDL), which was sufficient to establish the surgical indication; (2) Whole-body imaging excluded distant metastatic lesions, and there was no indication for preoperative neoadjuvant therapy (which requires biopsy to confirm histological subtype and guide treatment); (3) Given the tumor’s giant size and superficial location relative to the abdominal wall, biopsy was deferred to avoid the potential risk of tumor cell seeding along the needle tract; (4) The decision was in line with the 2024 National Comprehensive Cancer Network (NCCN) guidelines for soft tissue sarcoma: preoperative biopsy is not mandatory for resectable tumors with typical imaging features of WDL and no indication for neoadjuvant therapy.

#### Feasibility assessment of nephron-sparing surgery

Preoperative multidisciplinary evaluation was performed to assess the feasibility of nephron-sparing surgery (NSS, tumor resection with perinephric fat removal while sparing the kidney). However, NSS was not feasible for this case based on the following comprehensive assessment: (1) The giant tumor (250 mm × 92 mm) was firmly adherent to the anterior capsule of the right kidney, with severe compression and malrotation of the renal hilum, which made it impossible to completely separate the tumor from the renal parenchyma while ensuring the integrity of the tumor capsule; (2) Preoperative imaging confirmed that the tumor’s blood supply was derived from small branches of the right renal artery, and NSS carried an extremely high risk of incomplete tumor resection and intraoperative capsule rupture (tumor rupture is an independent risk factor for postoperative recurrence); (3) The patient had a congenital duplex renal collecting system, which increased the difficulty of precise dissection of the renal pelvis and ureter during NSS, with a high risk of intraoperative urinary tract injury; (4) The patient’s contralateral left kidney had completely normal morphology and function, and en bloc resection of the tumor and right kidney could ensure R0 resection with no long-term risk of renal insufficiency.

#### Surgical approach selection and rationale

The retroperitoneal approach was selected for this operation based on preoperative comprehensive evaluation, with the following core considerations:

1. Oncological principle compliance: Preoperative contrast-enhanced MRI confirmed that the tumor was strictly confined to the retroperitoneal space, with no evidence of invasion into the intra-abdominal viscera, and only required en bloc resection of the ipsilateral kidney and partial adrenal gland. The retroperitoneal approach can directly access the lesion without entering the abdominal cavity, enabling complete en bloc total retroperitoneal fat dissection (TRFD) on the affected side without interference from intra-abdominal organs. Previous studies have confirmed that complete TRFD is an independent protective factor for reducing local recurrence of RPLS [4], which is the core oncological advantage of this approach over the transabdominal approach.
2. Safety and minimally invasive advantage over open surgery: For giant RPLS, open surgery has long been considered the standard approach, but it is associated with large incision, high intraoperative blood loss, long postoperative recovery time, and high incidence of incision-related complications. In this case, the robot-assisted retroperitoneal approach achieved an estimated intraoperative blood loss of only 50 mL, no intraoperative blood transfusion, and the patient was discharged on postoperative day 6. These outcomes are significantly better than the published clinical data of open surgery for giant RPLS (mean intraoperative blood loss 300–800 mL, mean postoperative hospital stay 10–14 days [1]), confirming the minimally invasive advantage of this approach.
3. Technical feasibility and risk control: This approach provides direct access to the tumor and renal hilum, avoiding mobilization of intra-abdominal organs, thus reducing the risk of visceral injury and postoperative intestinal complications. Preoperative imaging clearly showed that the tumor’s blood supply was derived from small branches of the renal artery, and the retroperitoneal approach allowed for early and precise control of the renal hilum vessels, which is critical for reducing intraoperative hemorrhage in giant tumor surgery.
4. Clear indication boundary: We also clarified that the transabdominal approach or open surgery is still the preferred option for cases with preoperative imaging highly suggesting extensive invasion of multiple intra-abdominal organs requiring complex multivisceral resection, or high-grade tumors with high risk of adjacent organ invasion. For this case strictly confined to the retroperitoneal space, the retroperitoneal approach can fully meet the oncological requirements of R0 resection. We fully acknowledge that preoperative imaging has inherent limitations in accurately distinguishing between fibrous adhesion and microscopic tumor invasion, so this approach is only applicable to cases strictly confirmed to be confined to the retroperitoneal space without suspected intra-abdominal organ invasion via comprehensive preoperative imaging.

#### Indication of retroperitoneal lymph node dissection

Preoperative contrast-enhanced MRI showed strip-like soft tissue density with tortuous veins in the right renal hilum and adrenal region, which could not completely exclude regional lymph node involvement. Although the overall incidence of lymph node metastasis in well-differentiated liposarcoma (WDL) is low, Brehat et al. [2] reported that the incidence of lymph node metastasis in giant RPLS (> 20 cm) was up to 12.3%, significantly higher than 2.1% in small RPLS (< 10 cm). Meanwhile, the 2024 NCCN Guidelines for Soft Tissue Sarcoma recommend regional lymph node evaluation for resectable soft tissue sarcoma to clarify the pathological stage. Therefore, we planned retroperitoneal lymph node dissection during the operation to clarify the pathological stage, exclude occult lymph node metastasis, and guide postoperative adjuvant treatment.

### Operative procedure

After ruling out surgical contraindications, we formulated the surgical plan of Da Vinci Xi Surgical System robot-assisted retroperitoneal tumor resection combined with right nephrectomy and retroperitoneal lymph node dissection via the retroperitoneal approach, and the operation was performed on August 6, 2024. The total operative time was 185 min, the estimated intraoperative blood loss was approximately 50 mL, and no intraoperative blood transfusion was required. Intraoperative exploration confirmed dense adhesion between the tumor and the anterior capsule of the right kidney, partial right adrenal gland, and ventral parietal peritoneum, with mild adhesion to the inferior vena cava wall. No intra-abdominal organ invasion was observed, which was fully consistent with preoperative imaging.

**Fig. 2:**
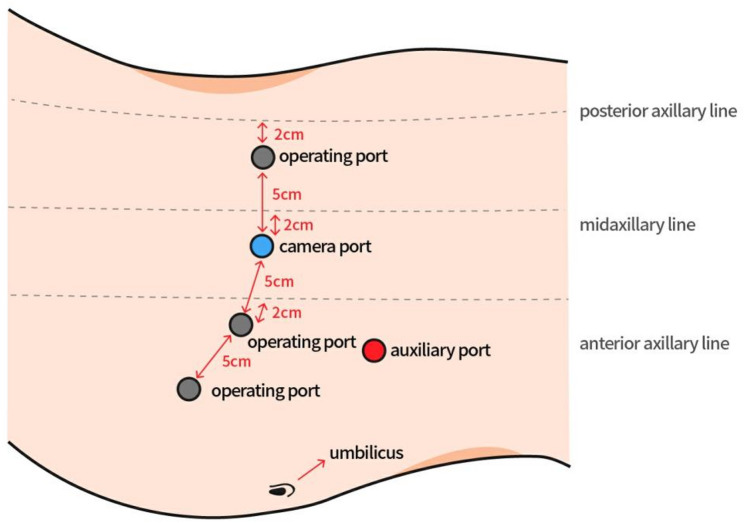
Schematic diagram of trocar placement for Da Vinci robotic surgery via the retroperitoneal approach. The patient was placed in the lateral decubitus position. The blue marker indicates the 12 mm optical camera port; gray markers indicate 8 mm robotic operating ports; the red marker indicates the 12 mm auxiliary/assistant port. Anatomical reference lines (posterior axillary line, midaxillary line, anterior axillary line), the 2 cm distance from the ports to the corresponding reference lines, and the 5 cm spacing between adjacent robotic ports are marked to avoid mechanical arm interference

Port placement was performed following a customized retroperitoneal robotic configuration for giant retroperitoneal liposarcoma, with a standardized 5 cm oblique interval between adjacent robotic ports and a uniform 2 cm ventral offset to the corresponding anatomical reference lines, to avoid the “chopstick effect” of mechanical arm interference. The configuration is fully consistent with the anatomical landmarks and spatial layout shown in Fig. 2 (operative schematic diagram, with the patient in left lateral decubitus position):

After induction of general anesthesia, the patient was placed in a left lateral decubitus position with the waist elevated to fully expand the right lumbocostal space and retroperitoneal workspace. An initial retroperitoneal space was established using a balloon dilator: a small incision was made at the intersection of the posterior axillary line and the inferior margin of the 12th rib, followed by blunt dissection of the muscular layer and lumbodorsal fascia to access the retroperitoneal space. A balloon dilator was inserted and inflated with 600 mL of air, maintained for 5 min to expand the space and achieve compression hemostasis.

Subsequent trocars were placed under direct endoscopic visualization, with a standardized 5 cm oblique interval between adjacent robotic ports to avoid the “chopstick effect” of mechanical arm interference, with the specific configuration fully matching the schematic diagram as follows:

1. Robotic Operating Port 1 (8 mm robotic trocar, gray marker in the schematic): The first placed robotic port, located 2 cm ventral to the posterior axillary line (the dorsal-most horizontal anatomical reference line in the diagram), at the same level of the initial incision for retroperitoneal space establishment. It was docked to the first mechanical arm of the Da Vinci Xi Surgical System, equipped with Maryland dissecting forceps for precise tissue retraction, anatomical separation of the upper pole of the kidney and adrenal gland, and fine dissection around the great vessels.
2. Camera Port (12 mm optical trocar, blue marker in the schematic): Placed 5 cm caudal and ventral to Robotic Operating Port 1, 2 cm ventral to the midaxillary line (the middle horizontal anatomical reference line in the diagram). It was used to accommodate the 3D endoscopic camera of the Da Vinci Xi Surgical System for full intraoperative magnified visualization of the entire retroperitoneal operative field, with pneumoperitoneum pressure maintained at 12–14 mmHg throughout the procedure.
3. Robotic Operating Port 2 (8 mm robotic trocar, gray marker in the schematic): Placed 5 cm caudal and ventral to the camera port, 2 cm ventral to the anterior axillary line (the ventral-most horizontal anatomical reference line in the diagram). It was docked to the second mechanical arm of the system, equipped with an ultrasonic scalpel for core tissue dissection, vascular control, and division of the renal hilum vessels and tumor feeding arteries.
4. Robotic Operating Port 3 (8 mm robotic trocar, gray marker in the schematic): Placed 5 cm caudal and ventral to Robotic Operating Port 2, at the caudal side of the operative field. It was docked to the third mechanical arm of the system, equipped with ProGrasp forceps for multi-angle operative field exposure, traction of the giant tumor capsule, and assistance for standardized retroperitoneal lymph node dissection at the distal abdominal aorta and iliac vessels.
5. Auxiliary/Assistant Port (12 mm assistant trocar, red marker in the schematic): Placed at the same horizontal (craniocaudal) level as Robotic Operating Port 2, on the ventral side of the anterior axillary line, to accommodate the assistant’s instruments. It was used for intraoperative suction, Hem-o-lok clip application, auxiliary irrigation, and specimen retrieval, with position adjustable according to intraoperative manipulation requirements.

After confirming no mechanical arm collision or instrument interference between all ports, and the retroperitoneal space was fully expanded with clear visualization of the perirenal fascia, tumor capsule margin, and adjacent anatomical structures under the 3D endoscopic view, the robotic system was docked, and the formal tumor dissection and nephrectomy procedure was initiated.

**Fig. 3:**
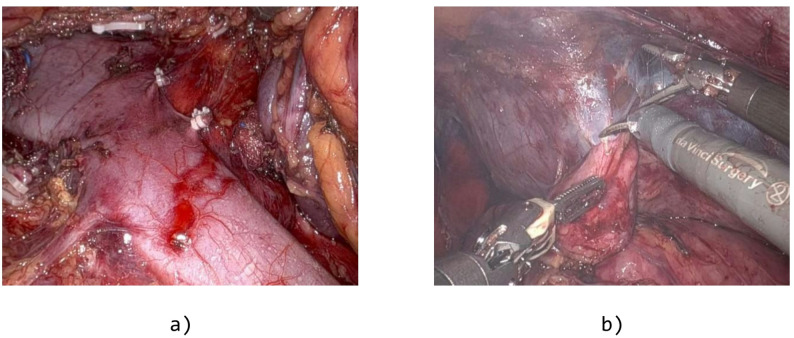
Illustrative images of key intraoperative procedures. (a) Intraoperative demonstration of en bloc dissection of the tumor-adherent parietal peritoneum, which was completely included in the final intact resection specimen (b) Blunt dissection of the tumor-adherent parietal peritoneum during en bloc resection, (the peritoneum was included in the final en bloc specimen

**Fig. 4:**
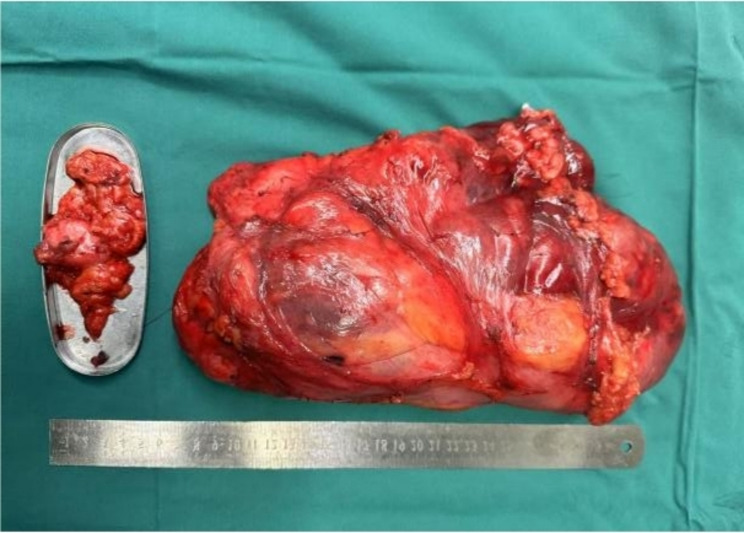
Postoperative specimen demonstrating the large tumor mass with surrounding retroperitoneal adipose tissue, right kidney, and tumor-adherent parietal peritoneum after complete en bloc surgical resection

Upon opening the perirenal fascia at the lower pole of the right kidney, the retroperitoneal mass was immediately visualized and dissected along its capsule. After identifying the inferior vena cava (IVC), dissection proceeded superiorly and inferiorly along the interface between the vena cava and the mass. The mass was observed compressing the renal hilum. At the level of the right renal hilum, the main trunk of the right renal artery was first identified at its origin from the abdominal aorta, ligated with three Hem-o-lok clips at the proximal end, and transected. Subsequently, dissection continued inferiorly along the mass capsule to its lower margin at the right iliac fossa. After meticulous separation, additional small arterial branches supplying the tumor, which originated from the distal segment of the transected right renal artery (the side towards the kidney and tumor), were identified. Even after the main trunk of the right renal artery was transected, these small branches still had potential retrograde blood supply from the perirenal collateral circulation (including lumbar artery and adrenal artery branches). Failure to ligate these branches would lead to intraoperative oozing or delayed postoperative hemorrhage, so they were ligated with three Hem-o-lok clips and transected to ensure complete hemostasis. Simultaneously, the right ureter was dissected along the ventral aspect of the right kidney, whose normal anatomical structure was altered due to tumor compression, ligated with two Hem-o-lok clips, and transected. The right renal vein was identified at the upper-middle pole of the right kidney, ligated with three Hem-o-lok clips, and subsequently transected. During superior dissection along the dorsal aspect, significant adhesions between the mass and the right adrenal gland were encountered. The central vein of the right adrenal gland was ligated with three Hem-o-lok clips prior to partial adrenalectomy. At the junction between the mass and liver, dissection proceeded ventrally, requiring careful blunt dissection to maintain the integrity of the tumor capsule. Dissection continued until the mass was completely mobilized, with the integrity of the tumor capsule strictly maintained throughout the process. Upon confirmation of complete tumor resection and negative margins, multiple titanium metal clips were placed at the tumor bed (including the right renal fossa, the base of the resection area, the margin of the partial adrenal gland resection, and the high-risk area of potential residual tumor) to provide precise localization for potential subsequent adjuvant radiotherapy or salvage radiotherapy in case of recurrence, which is fully compliant with the 2024 NCCN Guidelines for Soft Tissue Sarcoma. Bipolar electrocoagulation was performed for hemostasis at the surgical margins (Fig. 3). The laparoscopic port was then enlarged, and the specimen was retrieved intact via hand assistance. A single silicone drainage tube was placed adjacent to the right renal fossa (Fig. 1). The mass, measuring 25 × 15 cm, was completely resected and removed intact (Fig. 4).

### Postoperative pathological findings

The surgical specimen was subsequently sent for pathological examination in strict accordance with the 2024 NCCN Soft Tissue Sarcoma Guidelines. Pathology examination confirmed that the tumor was a well-differentiated LS. The fresh intraoperative specimen measured 25 × 15 × 8 cm, and the formalin-fixed paraffin-embedded specimen measured 25 × 12.5 × 3.5 cm (adipose tissue typically undergoes 30%-50% shrinkage in the anterior-posterior diameter after formalin fixation, which was fully consistent with the preoperative MRI measurement of 250 mm×92 mm). No tumor invasion was found in the adjacent right kidney, right adrenal gland, or peritoneum.

We performed standardized full circumferential margin assessment for the specimen: the entire specimen was sampled at 6 circumferential margins (ventral, dorsal, cranial, caudal, medial, lateral), and all marginal tissues were evaluated microscopically. All 6 circumferential surgical margins were microscopically negative for tumor cells, with the narrowest margin width of 2 mm, confirming that R0 resection was successfully achieved.

En bloc resection scope: The operation was performed in strict accordance with the en bloc resection principle for retroperitoneal sarcoma, and the specimen was resected in one intact piece, with no piecemeal resection performed during the entire operation. The en bloc specimen included: ①the entire retroperitoneal tumor with intact capsule; ②the right kidney and complete ipsilateral Gerota fascia and perirenal fat; ③the partial right adrenal gland with tumor adhesion; ④ the parietal peritoneum adherent to the ventral side of the tumor. The integrity of the tumor capsule was strictly maintained throughout the entire dissection process, and all tumor-adherent tissues were completely included in the en bloc specimen, which fully complied with the oncological principles of retroperitoneal sarcoma resection. No piecemeal resection or intracapsular dissection was performed during the entire operation, and all tumor-adherent tissues were resected en bloc with the primary tumor, which fully complies with the oncological principles of retroperitoneal sarcoma resection.

Retroperitoneal lymph node dissection results: A total of 12 regional lymph nodes were dissected, including 4 lymph nodes in the renal hilum, 5 in the paracaval region, and 3 in the para-aortic region. No tumor metastasis was found in any of the 12 lymph nodes, which excluded occult lymph node involvement. The final pathological stage was pT2bN0M0, G1 (well-differentiated), which provided a clear basis for postoperative adjuvant treatment decision-making.

**Fig. 5:**
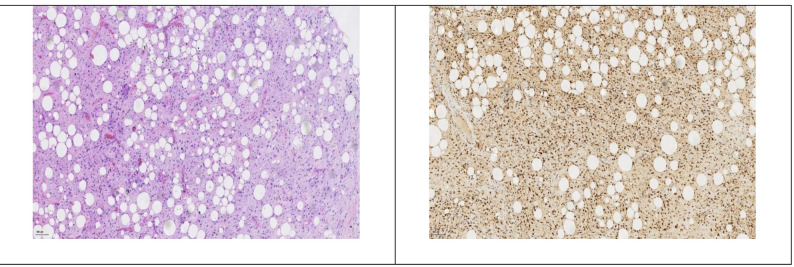
Histopathological and immunohistochemical findings of the tumor tissue

Immunohistochemistry and MDM2 fluorescence in situ hybridization (FISH) supported the diagnosis, yielding the following results: MDM2 (+), P16 (+), RB1 (partial +), CD34 (partial +), S-100 (+), PTEN (partial +), P53 (+) in 50%–60% of cells (weak to moderate intensity), Ki-67 (+) 10%–20%, CD117 (–), MelanA (–), HMB45 (–) (Fig. 5). The postoperative immunohistochemical result of MDM2 positive amplification in this case further confirmed the diagnosis of WDL, which was highly consistent with the preoperative imaging evaluation, and strongly supported our decision to forgo preoperative core needle biopsy in accordance with the 2024 NCCN guidelines. R0 resection, intact tumor capsule preservation and complete TRFD achieved in this operation are all core factors to reduce the risk of postoperative local recurrence, which lay a foundation for the good short-term oncological outcome of the patient.

### Postoperative course and follow-up

On the first postoperative day, serum creatinine (Scr) was 92 µmol/L, urinalysis was unremarkable, and ultrasonography demonstrated no left renal hydronephrosis. Pathological result timeline and MDT decision-making process:

- Postoperative day 3: Routine hematoxylin-eosin (HE) staining pathology returned a preliminary diagnosis of well-differentiated liposarcoma, with all circumferential surgical margins negative for tumor cells;
- Postoperative day 4: Immunohistochemistry (IHC) and MDM2 fluorescence in situ hybridization (FISH) results were fully issued, confirming the final diagnosis of WDL, consistent with the preliminary HE diagnosis;
- Postoperative day 5: A multidisciplinary team (MDT) consultation including urology, oncology, and pathology departments was convened to evaluate the patient’s recurrence risk and formulate adjuvant treatment plan.

Perioperative recovery was uneventful, and the patient was discharged on postoperative day 6.

After MDT evaluation, oral anlotinib was prescribed as adjuvant targeted therapy, with a regimen of 12 mg once daily (2 weeks on/1 week off, planned for 6 consecutive cycles). The indication for adjuvant therapy was based on the patient’s multiple high-risk factors for postoperative local recurrence, which were clearly defined in the MDT discussion: (1) Giant tumor with a maximum diameter of 25 cm, which has been confirmed as an independent risk factor for local recurrence of WDL, with a 5-year local recurrence rate of more than 30%; (2) Dense adhesion between the tumor and the renal capsule and peritoneum, even though no microscopic invasion was found in the final pathology; (3) Congenital duplex renal collecting system, which limited the scope of local dissection during the operation.

We acknowledge that current NCCN and ESMO guidelines do not recommend routine adjuvant chemotherapy or targeted therapy for completely resected WDL. This adjuvant treatment decision was fully individualized for this high-risk patient, supported by the following clinical evidence: (1) A phase 2, double-blinded, randomized controlled trial published in Clinical Cancer Research in 2025 confirmed that anlotinib as adjuvant therapy significantly improved disease-free survival in patients with high-risk localized soft tissue sarcomas (Wang et al. [5], cited in the reference list); (2) Multiple clinical studies have confirmed that anlotinib has definite anti-tumor activity in advanced and metastatic WDL, with a disease control rate of more than 60%, providing a pharmacological basis for its adjuvant application to reduce recurrence risk. This adjuvant targeted therapy was an individualized decision for this specific high-risk patient, and does NOT constitute a routine clinical recommendation for all patients with completely resected WDL, which is fully consistent with the current NCCN and ESMO guidelines that do not recommend routine adjuvant therapy for this population.

Follow-up CT scans at 3 months, 6 months, and 1 year postoperatively demonstrated no evidence of tumor recurrence, and no abnormalities were detected in serum creatinine (Scr), urinalysis, or other relevant laboratory examinations. The patient completed all 6 cycles of anlotinib adjuvant therapy as planned, with no grade 3 or above treatment-related adverse events during the treatment period.

## Discussion

Retroperitoneal liposarcoma (RPLS) is classified clinically into four main histological subtypes: well-differentiated liposarcoma (WDL), dedifferentiated liposarcoma (DDL), myxoid liposarcoma (MLS), and pleomorphic liposarcoma (PLS) [6]. Among these, WDL and DDL are the most commonly encountered types in clinical practice. Given its insidious growth pattern and deep retroperitoneal location, imaging studies are crucial for achieving accurate diagnosis and determining appropriate therapeutic strategies. In CT and MRI, RPLS typically presents with features such as thick septa, nodular or lobulated masses, non-adipose regions, and reduced proportions of fatty components within the lesion [7]. Typical WDL generally demonstrates a density similar to adipose tissue throughout most of the tumor, with irregularly distributed fibrous septa [8] and a well-defined, intact capsule. These tumors are often large and tend to displace adjacent organs and tissues without invasion or destruction of surrounding anatomical structures. In contrast, DDL typically presents as a solid mass containing variable amounts of low-density fatty components, exhibiting a clear demarcation between fatty and dedifferentiated regions. Calcification or ossification may occasionally be observed [9]. The capsule is often incomplete, and the tumor commonly shows significant adhesions to surrounding tissues. Less common subtypes, including MLS and PLS, generally have a low proportion of fatty components (usually < 25%) on imaging, thereby necessitating histopathological evaluation for definitive diagnosis. MLS primarily consists of round cells and lipoblasts, with the proportion of round cells correlating with tumor aggressiveness [10]. Conversely, PLS is characterized by marked cellular pleomorphism. Although areas containing mature adipocytes may exist, adipocyte sizes vary considerably. Lipoblasts or vacuolated cells are frequently observed within a limited myxoid stroma, and some tumor cells may feature multiple overlapping nuclei or form multinucleated giant cells with petal-like arrangements.

Surgical resection remains the primary treatment modality for RPLS [11–13], with the goal of achieving an R0 resection (microscopically negative margins). However, in clinical practice, attaining a true R0 resection is often challenging, and most procedures result in R1 resections, where microscopic residual tumor cells may persist at the resection margin. Currently, there is no clear consensus regarding the optimal extent of surgical resection for RPLS. Some researchers suggest that, for high-grade RPLS, extended resection, including potentially invaded adjacent organs, may improve long-term survival [14]. In contrast, for low-grade RPLS, expanding the surgical field does not significantly enhance prognosis and may instead increase postoperative complications [15]. For selected low-grade RPLS cases with small tumor volume, clear boundary with the renal parenchyma, no renal hilum invasion, and normal ipsilateral renal function, nephron-sparing surgery with complete resection of the tumor and perinephric fat can achieve good oncological outcomes while preserving renal function, which has been confirmed by previous clinical studies. Studies have shown that en bloc resection of the kidney, pancreas, surrounding fat, and adjacent myofascia during RPLS surgery significantly improves local recurrence-free survival (LRFS) [4–16]. Pathological analysis indicates that, among all RPLS nephrectomy specimens, only 6.1% of cases in contact with the kidney showed histological invasion, whereas colectomy specimens exhibited even higher invasion rates, with 46.9% demonstrating colonic or mesenteric involvement [2]. Among RPLS patients undergoing biopsy, 51.4% were preoperatively diagnosed as DDL on imaging, while 75.6% were confirmed as DDL on final histopathological examination. Thway and Khin reported that approximately 10% of WDL showed a tendency to dedifferentiate into DDL, displaying greater invasiveness, higher local recurrence rates, and increased metastatic potential [17]. At present, local recurrence remains the leading cause of death in RPLS. To improve long-term survival, extensive or compartmental resection should be considered the preferred surgical strategy. Recent studies further demonstrate that preoperative neoadjuvant radiotherapy, particularly for patients with DDL, significantly reduces the risk of postoperative local recurrence compared with surgery alone [18].

Compared with common epithelial-derived tumors, RPLS originates from mesenchymal tissue and often presents as a relatively large mass. The disease is frequently associated with adhesion to and invasion of surrounding organs, and open surgery has traditionally been the primary treatment approach. However, with the recent advances and adoption of laparoscopic and Da Vinci Xi Surgical System robotic surgical techniques, surgical management has become more versatile. The Da Vinci Xi Surgical System overcomes the limitations of conventional laparoscopy, such as unstable visual fields and restricted instrument maneuverability. It provides enhanced three-dimensional visualization and greater precision in dissecting tumors located within deep or complex retroperitoneal spaces. This system enables the complete and safe resection of retroperitoneal tumors adherent to major vessels, offering superior precision, safety, and stability compared to traditional laparoscopic approaches. Tumor rupture is an independent risk factor for RPLS recurrence [19]. The Da Vinci Xi Surgical System, through its highly precise instrumentation and superior control, effectively minimizes the risk of intraoperative tumor rupture or damage. Moreover, compared with traditional open surgery, it substantially reduces postoperative recovery time and the incidence of incision-related complications such as infection and delayed wound healing. However, the Da Vinci Xi Surgical System has certain limitations. In some clinical cases, robotic procedures must be converted to open surgery due to intraoperative challenges. Hao Q et al. reported [3] that tumor size ≤ 64 mm, well-defined tumor margins, absence of local invasion, and no requirement for combined organ resection are critical factors influencing the success of Da Vinci Xi Surgical System robotic surgery in treating RPLS. For RPLS, the core oncological goals of surgery are to achieve R0 resection, maintain the integrity of the tumor capsule, and complete total retroperitoneal fat dissection (TRFD). Tumor capsule rupture is an independent risk factor for postoperative local recurrence and distant metastasis of RPLS [19], and open surgery has a relatively high risk of capsule rupture due to limited visualization of the deep retroperitoneal space. The Da Vinci surgical system provides 10–15 times magnified 3D vision and ultra-fine operating instruments, which can achieve more precise sharp dissection along the tumor capsule, effectively reducing the risk of intraoperative capsule rupture, which is critical to improve the oncological outcome of patients.

Existing clinical studies on robot-assisted surgery for RPLS are mostly based on the transabdominal approach, which has a wider operative field and is more suitable for complex multivisceral resection. For the retroperitoneal approach, the largest case series (105 patients) reported by Hao Q et al. [3] confirmed that the critical factor for successful surgery was a tumor diameter ≤ 64 mm, and there are few reports on the application of this approach in giant RPLS (> 20 cm) worldwide. Da Vinci Xi Surgical System robotic laparoscopic surgery for RPLS is primarily performed via a transabdominal approach, as the abdominal cavity provides adequate space, reduces interference from robotic arms, and offers a wider operative view, facilitating surgical manipulation. We fully agree with the clinical consensus that the retroperitoneal approach is a widely accepted and mature surgical method for moderately sized retroperitoneal tumors (generally defined as maximum diameter < 10 cm), which has been confirmed by a large number of clinical studies including previous reports in BMC Urology. However, most existing clinical studies on robot-assisted retroperitoneal approach for RPLS focus on these moderately sized tumors, and there are very few reports on its application in giant RPLS (> 20 cm) like the present case (25 cm maximum diameter). Although the retroperitoneal approach provides a smaller operative field, its advantages include proximity to the tumor, avoidance of the abdominal cavity, and reduced interference from intra-abdominal viscera.

In the present case, the rationale for selecting the retroperitoneal approach was as follows: (1) Preoperative contrast-enhanced MRI confirmed that the tumor was strictly confined to the retroperitoneal space, with no evidence of invasion into the intra-abdominal viscera or intestinal loops, eliminating the need for extensive intra-abdominal exploration or multivisceral resection beyond the kidney and partial adrenal gland. We acknowledge that preoperative imaging has inherent limitations in accurately distinguishing between fibrous adhesion and microscopic tumor invasion. For cases where preoperative imaging highly suggests extensive invasion of multiple intra-abdominal organs requiring complex multivisceral resection, or for high-grade tumors with a high risk of adjacent organ invasion that may require extended en bloc resection, the transabdominal approach is more appropriate due to its wider operative field and greater flexibility for combined organ resection, which is consistent with previous studies on the survival benefit of combined adjacent organ resection for high-grade RPLS. Consistent with this view, the present case only required en bloc resection of the ipsilateral kidney and partial adrenal gland, with no preoperative evidence of gastrointestinal invasion, so the retroperitoneal approach could fully meet the surgical requirements while avoiding the shortcomings of the transabdominal approach. (2) The retroperitoneal approach provides direct access to the tumor and renal hilum, avoiding the need for mobilization of intra-abdominal organs, thus reducing the risk of visceral injury and postoperative intestinal complications. (3) This approach facilitates complete dissection of the ipsilateral retroperitoneal fat, which is associated with a lower risk of local recurrence. (4) Preoperative imaging clearly demonstrated the tumor’s blood supply was derived from small branches of the renal artery, allowing for early and precise control of the renal hilum vessels via the retroperitoneal route. Enhanced imaging modalities, such as computed tomography urography (CTU) and magnetic resonance imaging (MRI), confirmed the tumor’s precise location and its lack of invasion into intra-abdominal organs. Considering the tumor’s compression of the kidney and the presence of renal arterial supply demonstrated by imaging, the decision was made to remove both the tumor and ipsilateral kidney [4]. The specific intraoperative procedures of the retroperitoneal approach are detailed in the Case Presentation section. Briefly, this approach allowed us to directly access the renal hilum for early vascular control, dissect the tumor along the capsule to maintain its integrity, and complete en bloc resection of the tumor and ipsilateral kidney without intra-abdominal organ mobilization. We sincerely acknowledge that the retroperitoneal approach is widely accepted and mature for moderately sized retroperitoneal tumors such as renal cell carcinoma and adrenal tumors, which is consistent with clinical consensus. Meanwhile, our case further demonstrates the feasibility and safety of this approach in the treatment of large (> 20 cm) retroperitoneal liposarcoma with strict preoperative screening. For this case with the tumor strictly confined to the retroperitoneal space and no intra-abdominal organ invasion, the retroperitoneal approach can still achieve complete R0 resection with intact tumor capsule, while avoiding the interference of intra-abdominal viscera, which expands the applicable scope of the retroperitoneal approach for robotic RPLS surgery. Compared with the transabdominal approach for RPLS, the retroperitoneal approach significantly reduces the volume of tissue requiring dissection, thereby decreasing the risk of intra-abdominal organ injury. Additionally, compared with the transabdominal approach, the retroperitoneal approach can directly access the retroperitoneal space, facilitate complete en bloc total retroperitoneal fat dissection (TRFD) on the affected side without intra-abdominal organ interference. Previous studies have confirmed that complete TRFD is an independent protective factor for reducing local recurrence of RPLS [4], which is the core oncological advantage of this approach for RPLS. The customized trocar configuration in this case, with a uniform 1 cm ventral offset and 5 cm equal interval between adjacent ports, effectively avoids the “chopstick effect” of robotic arms during the operation. This configuration provides sufficient operative space for the dissection of the 25 cm giant tumor, precise control of the renal hilum vessels, and standardized retroperitoneal lymph node dissection via the retroperitoneal approach, which further ensures the safety and integrity of the en bloc resection.For selected low-grade RPLS cases with clear boundary with the renal parenchyma and no renal hilum invasion, nephron-sparing surgery is the preferred strategy to preserve renal function, which is fully consistent with existing clinical evidence.

Regarding the impact of surgical approach on resection margin status, we conducted an objective analysis based on this case and existing clinical evidence: (1) For RPLS with extensive intra-abdominal organ invasion requiring multivisceral en bloc resection, the transabdominal approach provides a wider operative field and more flexible maneuverability, which is more conducive to achieving negative surgical margins and remains the recommended approach for such cases; (2) For this case, the tumor was strictly confined to the retroperitoneal space, with only ventral peritoneal adhesion and no intra-abdominal organ invasion. The retroperitoneal approach allowed us to completely resect the tumor-adherent peritoneum within the en bloc scope, and finally achieved R0 resection with negative margins, which is oncologically equivalent to the transabdominal approach for strictly selected cases; (3) We acknowledge that the transabdominal approach has inherent advantages in dealing with unexpected intraoperative tumor invasion of abdominal organs, but for preoperatively strictly screened cases confined to the retroperitoneal space, the retroperitoneal approach can achieve satisfactory oncological margins while avoiding intra-abdominal organ interference.

Regarding postoperative treatment for RPLS, the National Comprehensive Cancer Network (NCCN) guidelines have not recommended routine adjuvant chemotherapy for soft tissue sarcomas since 2017. Both the European Society for Medical Oncology (ESMO) and NCCN guidelines suggest considering neoadjuvant chemotherapy after confirming tumor subtype via biopsy [20–21]. For advanced or metastatic RPLS, a regimen comprising doxorubicin (ADM) combined with ifosfamide (IFO) remains the mainstay of treatment, though careful consideration of efficacy versus toxicity is essential. The NCCN guidelines also highlight targeted therapies (e.g., larotrectinib and pazopanib) and immunotherapy (e.g., pembrolizumab) for specific subtypes. For recurrent RPLS, surgical resection remains the preferred therapeutic approach. However, combining regional hyperthermia (RHT) with etoposide, ifosfamide, and doxorubicin chemotherapy significantly improves local tumor control; thus, RHT may represent an alternative treatment option to radical surgery [22]. In recent years, ongoing research into RPLS has led to the emergence of various combination therapeutic strategies, including chemotherapy combined with targeted therapy, chemotherapy combined with immunotherapy, targeted therapy combined with immunotherapy, and combinations of chemotherapy, targeted therapy, and immunotherapy. These novel approaches offer valuable perspectives for the management of RPLS. Follow-up recommendations suggest visits every 3–4 months for the first two years, every 6 months for the subsequent three years, and annually thereafter [23]. The case confirms the feasibility and safety of robot-assisted retroperitoneal resection for strictly selected giant RPLS (> 20 cm) compressing the kidney, with favorable short-term oncological outcomes. This approach may provide a technical reference and management strategy for selected patients with similar clinical characteristics.

### Clinical significance and innovation

The innovation and clinical value of this case, which are highly aligned with the readership scope of BMC Urology (urologists), are mainly reflected in the following three aspects:

1. Establishment of the first standardized urological decision-making pathway for RPLS compressing the kidney: Existing studies on RPLS are mostly conducted from the perspective of soft tissue sarcoma surgery, and there is no standardized, replicable decision-making pathway for urologists who are often the first to diagnose and manage RPLS compressing the kidney. From the urological management perspective, we systematically elaborated a complete, guideline-based decision-making process for such diseases, including: ① Preoperative biopsy decision-making criteria based on 2024 NCCN guidelines; ② NSS feasibility assessment system for RPLS involving the kidney; ③ Surgical approach selection criteria based on tumor anatomical scope. This pathway can be directly replicated in daily urological clinical practice, filling the gap of standardized urological management for such diseases, which is the core clinical value for the readers of BMC Urology.
2. Breakthrough of the technical indication limit of the retroperitoneal approach: To the best of our knowledge, this is the first published case worldwide confirming the feasibility and safety of robot-assisted retroperitoneal approach for giant RPLS (> 20 cm) strictly confined to the retroperitoneal space. Existing clinical studies on this approach for RPLS are all limited to moderately sized tumors (diameter < 10 cm), and the largest published case series (105 patients) confirmed that the critical threshold for successful surgery was a maximum tumor diameter ≤ 64 mm [3]. This case breaks the existing consensus on the indication limit of this approach, and we further clarified the strict indication criteria and contraindications, providing a feasible minimally invasive technical reference for strictly selected giant RPLS cases.
3. Verification of the oncological safety of the retroperitoneal approach for giant RPLS: This case confirmed that the robot-assisted retroperitoneal approach can simultaneously achieve three core oncological goals for giant RPLS: R0 resection with negative margins, complete preservation of the tumor capsule, and standardized TRFD. All these factors have been confirmed as independent protective factors for reducing local recurrence of RPLS [4]. This study provides a reliable minimally invasive surgical option for strictly selected giant RPLS cases, with favorable short-term oncological outcomes.

## Conclusion

Our findings are consistent with Lyu et al.’s report [24] in BMC Urology, which demonstrated that the retroperitoneal approach yields favorable therapeutic outcomes in the treatment of renal tumors located at different sites.

Compared with previous published series focusing on moderately sized retroperitoneal tumors, our case further breaks through the tumor size limit of the robot-assisted retroperitoneal approach, preliminarily confirms its feasibility and safety in giant RPLS (> 20 cm) strictly confined to the retroperitoneal space, and provides a standardized urological management reference for urologists managing similar cases.

### Limitations of this study

This study has several clear limitations:

1. This is a single-center, single-case report, only included the well-differentiated liposarcoma (WDL) subtype, and cannot be generalized to high-grade RPLS subtypes such as dedifferentiated liposarcoma.
2. The follow-up period of this case is short, and only short-term oncological outcomes were observed. The long-term oncological efficacy and safety of this approach still need to be verified by large-sample, multi-center, long-term follow-up prospective studies. Long-term follow-up (≥ 5 years) of this patient is ongoing, and the long-term outcomes will be reported in subsequent studies.
3. The indication of this approach is strictly limited to giant RPLS confined to the retroperitoneal space without intra-abdominal organ invasion, and cannot be generalized to all RPLS cases. For most RPLS cases requiring multivisceral resection, open surgery or transabdominal approach remains the standard preferred option.

## Supplementary Information

Supplementary Material 1

## Data Availability

The datasets generated and/or analysed during the current study are not publicly available due to the need to protect patient privacy (in line with the ethical approval requirements of the Ethics Committee of the Affiliated Hangzhou First People’s Hospital) but are available from the corresponding author (Yuyong Wang, E-mail: wangyuyong2003@163.com) on reasonable request.

## Abbreviations

RAL: Robot-assisted Laparoscopic Nephrectomy
RPLS: Retroperitoneal liposarcoma
WDL: Well-differentiated liposarcoma
DDL: Dedifferentiated liposarcoma
MLS: Myxoid liposarcoma
PLS: Pleomorphic liposarcoma
Scr: Serum creatinine
CTU: Computed tomography urography
MRI: Magnetic resonance imaging
R0 resection: Resection with microscopically negative surgical margins
R1 resection: Resection with microscopically residual tumor at the surgical margin
TRFD: Total retroperitoneal fat dissection
NCCN: National Comprehensive Cancer Network
ESMO: European Society for Medical Oncology
ADM: Doxorubicin
IFO: Ifosfamide
RHT: Regional hyperthermia
Trocar: Disposable laparoscopic puncture device for robotic and laparoscopic surgery
