# Supplementary material for: Robot-assisted nephrectomy for retroperitoneal liposarcoma compressing the kidney: a case report with urological management perspective
Source: BMC Urol. 2026 May 18;26:160. doi: 10.1186/s12894-026-02186-2 (PMC13371255; doi:10.1186/s12894-026-02186-2)
Supplement: Supplementary file 1 — Supplementary Material 1 [file 12894_2026_2186_MOESM1_ESM.pdf]

CARE
case report guidelines

CARE Checklist of information to include when writing a case report

(cc) BY-NC-ND

Topic	Item	Checklist item description	Reported on Line
**Title**	1	The diagnosis or intervention of primary focus followed by the words “case report”	Line22-26
**Key Words**	2	2 to 5 key words that identify diagnoses or interventions in this case report, including "case report"	Line44-45
**Abstract (no references)**	3a	Introduction: What is unique about this case and what does it add to the scientific literature?	Line37-43
	3b	Main symptoms and/or important clinical findings	Line76-81
	3c	The main diagnoses, therapeutic interventions, and outcomes	Line22-36
	3d	Conclusion—What is the main “take-away” lesson(s) from this case?	Line587-594
**Introduction**	4	One or two paragraphs summarizing why this case is unique (**may include references**)	Line557-585
**Patient Information**	5a	De-identified patient specific information.	Line76-88
	5b	Primary concerns and symptoms of the patient.	Line76-83
	5c	Medical, family, and psycho-social history including relevant genetic information	Line80-81
	5d	Relevant past interventions with outcomes	Line83-85
**Clinical Findings**	6	Describe significant physical examination (PE) and important clinical findings.	Line81-83
**Timeline**	7	Historical and current information from this episode of care organized as a timeline	Line76-88
**Diagnostic Assessment**	8a	Diagnostic testing (such as PE, laboratory testing, imaging, surveys).	Line85-96
	8b	Diagnostic challenges (such as access to testing, financial, or cultural)	N/A
	8c	Diagnosis (including other diagnoses considered)	Line94-96
	8d	Prognosis (such as staging in oncology) where applicable	Line365-369
**Therapeutic Intervention**	9a	Types of therapeutic intervention (such as pharmacologic, surgical, preventive, self-care)	Line181-189
	9b	Administration of therapeutic intervention (such as dosage, strength, duration)	Line181-189
	9c	Changes in therapeutic intervention (with rationale)	N/A
**Follow-up and Outcomes**	10a	Clinician and patient-assessed outcomes (if available)	Line365-369
	10b	Important follow-up diagnostic and other test results	Line365-369
	10c	Intervention adherence and tolerability (How was this assessed?)	Line365-369
	10d	Adverse and unanticipated events	N/A
**Discussion**	11a	A scientific discussion of the strengths AND limitations associated with this case report	Line586-608
	11b	Discussion of the relevant medical literature **with references**.	Line663-723
	11c	The scientific rationale for any conclusions (including assessment of possible causes)	Line587-594
	11d	The primary “take-away” lessons of this case report (without references) in a one paragraph conclusion	Line590-594
**Patient Perspective**	12	The patient should share their perspective in one to two paragraphs on the treatment(s) they received	N/A
**Informed Consent**	13	Did the patient give informed consent? Please provide if requested	Yes ☑ No ☐
